# Curcumin protects PC12 cells from a high glucose-induced inflammatory response by regulating the miR-218-5p/TLR4 axis

**DOI:** 10.1097/MD.0000000000030967

**Published:** 2022-10-07

**Authors:** Yuan Cui, Hong-Tao Song, Pei Zhang, Xiao Yin, Ying Wang, Xuan Wei, Xin-Ju Jia

**Affiliations:** a Department of Neurology, Affiliated Hospital of Hebei Academy of Traditional Chinese Medicine, Shijiazhuang, Hebei Province, China; b Department of Vascular surgery, Shijiazhuang Second Hospital, Shijiazhuang, Hebei Province, China; c Department of Diabetes Screening Centre, Shijiazhuang Second Hospital, Shijiazhuang, Hebei Province, China; d Department of Traditional Chinese Medicine, Shijiazhuang Yuxi Community Health Service Center, Shijiazhuang, Hebei Province, China; e Department of Hemodialysis, Shijiazhuang Second Hospital, Shijiazhuang, Hebei Province, China; f Department of Endocrinology, Shijiazhuang Hospital of Traditional Chinese Medicine, Shijiazhuang, Hebei Province, China; g Department of Endocrinology, The First Hospital of Hebei Medical University, Shijiazhuang, Hebei Province, China.

**Keywords:** curcumin, diabetic encephalopathy, inflammation, miRNAs, TLR4

## Abstract

Curcumin exerts a protective effect on diabetic encephalopathy (DN), It is known for its potent neuroprotective, anti-inflammatory, antioxidant, and anticancer properties. However, the underlying mechanisms of curcumin’s neuroprotective effects resulting from high glucose (HG)-induced injuries remain unknown. The purpose of this study is to identify the protective mechanism of Curcumin in the DN.

**Methods:**

In this study, pheochromocytoma cells (PC12 cells) were pretreated with different concentrations of Curcumin and then co-treated with Curcumin and glucose for 48 hours, and the cell viability was evaluated by CCK-8, the expression of the inflammatory mediators were detected by ELISA, the miR-218-5p and toll-like receptors (TLR4) level were examined by both quantitative real-time polymerase chain reaction (qRT-PCR) and Western blotting, the potential target genes of miR-218-5p were identified using luciferase reporter assay.

**Results:**

The viability of PC12 cells treated with HG was significantly reduced in a dose- and time-dependent manner. Cotreatment of curcumin with HG significantly increased cell viability. Curcumin inhibited the expression of the inflammatory mediators, tumor necrosis factor-α (TNF-α) and interleukin 6 (IL-6), and induced the expression of the anti-inflammatory mediator interleukin-10 (IL-10). Curcumin upregulated the levels of miR-218-5p and downregulated the expression of TLR4 in HG-treated PC12 cells. The curcumin-induced anti-inflammatory effect was abrogated by a miR-218-5p inhibitor and overexpression of TLR4. The results suggest that curcumin ameliorates the inflammatory response by upregulating miR-218-5p levels in PC12 cells.

**Conclusions:**

Our results indicate a protective role for curcumin in PC12 cells and suggest that it should be considered for the prophylactic treatment of DN in the future.

## 1. Backgrounds

Diabetic encephalopathy (DN) is a major complication of diabetes and is associated with high morbidity rates.^[1]^ Neurodegenerative disorders, such as impaired learning ability, memory, and intelligence, are common characteristic of DN, which are caused by age of onset and the duration of diabetes.^[2]^ Although the incidence of DN is increasing rapidly, the underlying mechanisms are not fully understood and effective treatment strategies for this disease are lacking. Recently, reports have focused on multiple factors that are involved in the development and progression of DN, including oxidative stress and apoptosis.^[3,4]^ In addition, inflammatory mechanisms play an important role in the pathogenesis and progression of DN, which may aggravate DN symptoms and increased the mortality of patients with DN.^[5,6]^ Thus, we hypothesize that inhibiting the inflammatory response may represent a therapeutic strategy to ameliorate neural damage associated with diabetes.

Curcumin is a promising natural ingredient, which is extracted from turmeric (Curcuma longa). It is known for its potent neuroprotective, anti-inflammatory, antioxidant, and anticancer properties.^[7–11]^ Several studies have demonstrated that curcumin plays a protective role in several neurodegenerative diseases, including Parkinson’s disease,^[12]^ Alzheimer’s disease,^[13]^ and Huntington’s disease,^[14]^ as well as neuroinflammation.^[15]^ Many studies have described the anti-inflammatory effects of curcumin in nervous system disorders, chronic inflammation, cancer, cardiovascular disease, and other common diseases. Furthermore, a recent study showed that curcumin repressed PC12 cell death by inhibiting the AKT/mTOR signaling pathway, indicating the potential of curcumin for the treatment of Parkinson’s disease.^[16]^ However, the underlying mechanisms of curcumin’s neuroprotective effects resulting from high glucose-induced injuries remain unknown.

MicroRNAs (miRNAs) with a length of 21 to 25 nucleotides bind to their target genes at the 3’-untranslated region (3’UTR) to inhibit translation or degrade messenger RNA transcripts.^[17]^ The function of miRNAs is closely associated with neuroinflammation, microglial activation, and apoptosis, and they may be involved in the pathogenesis of many diseases including neurological diseases.^[18,19]^ Perdoncin et al recently identified differentially expressed miRNAs in obesity that were associated with cognitive decline and neurodegenerative disorders This suggests a potential link between miRNAs levels and obesity-associated cognitive decline and neurodegeneration.^[20]^ Recent studies have shown that microRNA-218 (miR-218) is associated with several cognition-related neurodegenerative and neuropsychiatric disorders.^[21]^ In the brain, miR-218 is highly expressed in astrocytes and thus impacts gliomagenesis. miR-218 is also abnormally expressed in a variety of cognition-related neurodegenerative and neuropsychiatric disorders; however, it remains unclear whether miR-218 plays a direct role in diabetic encephalopathy. It has been suggested that curcumin can alleviate oxidative damage by regulating the miR-1287-5p/LONP2 axis in SH-SY5Y cells.^[22]^ Based on the evidence above, curcumin may exhibit a neuroprotective effect by regulating miR-218-5p in the high glucose (HG)-induced inflammatory response in DN.

In this study, we determined the protective effects of curcumin on the high glucose-induced cell inflammatory response in cultured pheochromocytoma cells (PC12 cells), which were derived from a pheochromocytoma of the rat adrenal medulla. This model is frequently used to study the underlying mechanisms of glucose neurotoxicity.^[23]^ We further examined whether curcumin could attenuate the high glucose-induced release of inflammatory factors by regulating miR-218-5p to identify new strategies for treating DN at the molecular level.

## 2. Methods

Ethical approval was not necessary because no patients’ information was collected according to Ethics Committee of affiliated hospital of Hebei University (Baoding, China).

### 2.1. Materials

Dulbecco’s modified eagle medium (DMEM) and fetal bovine serum (FBS) were purchased from Corning Life Sciences. Curcumin (cat# HY-N0005, greater than 96.0%) was purchased from MedChemExpress (MCE, New Jersey), dissolved in DMSO, and diluted with culture medium for cell experiments. Trizol reagent, PrimeScript RT reagent kit, and the PCR reagent kit were obtained from TaKaRa (cat# RR036A, 640210, TaKaRa Biotechnology, Dalian, China). The primers were synthesized by Invitrogen Life Tech (Carlsbad, CA). Protease inhibitors were purchased from Millipore Sigma. An ECL chemiluminescence detection kit (SuperSignal HRP) was purchased from Pierce (cat# 46640, ThermoFisher Scientific, Inc.).

### 2.2. Cell culture and high glucose treatment

Pheochromocytoma cells (PC12 cells) were purchased from the ATCC and cultured in DMEM containing 10% (V/V) FBS, 100 μg/mL streptomycin, and 100 units/mL penicillin at 37°C in 5% CO_2_ and 95% N_2_. PC12 cells were passaged every 2 days. The cells were seeded in DMEM containing 10% FBS and incubated for 24 hours. The medium was changed to DMEM containing 0.5% FBS for a 24 hours starvation period. The PC12 cells were then incubated in normal glucose (control group, 5 mM) for 24 hours, (HG group, 50–150 mM) for 24 hours, and treated with curcumin (1–10 μM) 2 hours before high glucose treatment for 48 hours.

### 2.3. PC12 cells transfection

PC12 cells were seeded randomly into 6-well plates. After the medium was replaced with serum-free DMEM, the PC12 cells were transfected with miR-NC (100 nM), miR-218-5p mimic (100 nM), miR-218-5p inhibitor (100 nM), pcDNA3.1 (1 μg), pcDNA3.1-TLR4 (1 μg) using Lipofectamine 2000 (cat# 11668-019, ThermoFisher Scientific, Inc.) according to the manufacturer’s instructions. The miR-218-5p mimic, miR-NC, miR-218-5p inhibitor, pcDNA3.1, and pcDNA3.1-TLR4 were obtained from Biomics Biotechnologies Co., Ltd. The culture medium was replaced with fresh DMEM containing 10% FBS following a 6-hours incubation period. After incubating for an additional 48 hours, Western blot analysis and quantitative real-time polymerase chain reaction (qRT-PCR) were used to determine the efficiency of transfection.

### 2.4. Cell proliferation assay

The effect of curcumin and HG on the proliferation of PC12 cells was determined by a CCK-8 assay (cat# CK04, Dojindo, Japan) based on the manufacturer’s protocol. Briefly, after treatment, the cells were cultured in a 96-well plate at a density of 2 × 10^3^ cells/well for 24 hours. The CCK-8 solution was added to each well and incubated at 3°C in the dark for 1 hour. The optical density values were detected at a wavelength of 450 nm.

### 2.5. Western blot analysis

Total cellular protein was extracted using a radioimmunoprecipitation assay lysis buffer (50 mM Tris, pH 7.4, 150 mM NaCl, 1% Triton X-100, 1% sodium deoxycholate, 0.1% SDS, sodium orthovanadate, sodium fluoride, EDTA, and leupeptin) supplemented with a protease inhibitor cocktail (cat. nos. P0013B, Beyotime, Jiangsu, China). The concentration of the protein in the different groups was measured using a BCA protein assay kit (cat. nos. P0009, Beyotime, Jiangsu, China). Then, 30 μg of protein from each group were separated by SDS page gel electrophoresis and transferred to PVDF membranes (cat# 162-0177, Bio-Rad, Hercules, CA). The membranes were blocked with 5% skim milk powder in TBS containing 0.1% tris buffered saline with tween-20 (TBST) for 90 minutes at room temperature, followed by incubation with TLR4 (dilution, 1:1000; cat# Ab22048, Abcam) and GAPDH (dilution, 1:1000; cat# 8245, Abcam) antibodies overnight at 4°C. The next day, the blots were washed with TBST 5 times for 30 minutes and incubated with secondary antibodies (dilution, 1:20000; cat# ab288151, Abcam) for 90 minutes. The membranes were washed with TBST five times. An imaging system (Li-Cor; Lincoln, NE) was used to detect and analyze the density of each band.

### 2.6. Real-time polymerase chain reaction analysis

Total RNA was isolated using an RNA extraction kit (cat# 28306, QIAGEN, Germany) following the manufacturer’s protocol. The quality (OD_260_/OD_280_ = 1.8–2.0) and concentration of the RNA from each group were assessed using a NanoDrop2000 (ThermoFisher Scientific, Inc.). RNA contamination and degradation were assessed on 1% agarose gels. Next, 1000 ng of total RNA was reverse transcribed into cDNA with the PrimeScript™ RT Master Mix (cat# RR036A, Takara, Dalian, China). The real-time PCR reactions were run in triplicate using SYBR green PCR Master Mix (cat# 640210, Takara, Dalian, China). The amplification reaction was conducted as follows: 5 seconds at 95°C, 20 seconds at 63.5° C, and 10 seconds at 72°C for 40 cycles. The amplification efficiency was 95.6%, and the relative mRNA expression level of the target gene was calculated by the 2^−ΔΔCT^ method. The sequences of the forward and reverse primers for the target genes are listed in Table 1.

**Table 1:** Primer sequences used for Real-time PCR.

Gene name	Sequences 5’–3’
TNF-α F	CAACCAACAAGTGATATTCTCCATG
TNF-α R	GATCCACACTCTCCAGCTGCA
IL-6 F	ACTTCCATCCAGTTGCCTTCTTGG
IL-6 R	TTAAGCCTCCGACTTGTGAAGTGG
IL-10 F	AGTCAACTACAAGCCCCACG
IL-10 R	GCAGCTTGTCCAGGGATTCT
GAPDH F	GCATCTTCTTGTGCAGTGCC
GAPDH R	GATGGTGATGGGTTTCCCGT
miR-218-5p F	ACACTCCAGCTGGGTTGTGCT
miR-218-5p F	TGTCGTGGAGTCGGCAATTC

F = forward, IL-6 = interleukin 6, IL-10 = interleukin-10, R = reverse, TNF-α = tumor necrosis factor-α.

### 2.7. Luciferase reporter assay

The potential target genes of miR-218-5p were identified using the Targetscan database. Among these candidates, TLR4 was selected because of its protective effect on neuronal cell inflammation. To determine the miR-218-5p binding site on the 3′-UTR of TLR4, a luciferase reporter plasmid, pMIR-TLR4-3′-UTR wild type (Wt), and pMIR-TLR4-3′-UTR mutant (Mut) were synthesized and co-transfected into PC12 cells along with miR-218-5p mimics or miR-NC in 96-well plates using Lipofectamine 2000. The luciferase activities were detected at 48 hours after transfection and were normalized to Renilla luciferase activity using the Dual-Luciferase reporter assay system (cat. Nos. E1980, Promega).

### 2.8. Enzyme linked immunosorbent assay

The concentrations of tumor necrosis factor-α (TNF-α), interleukin 6 (IL-6), and interleukin-10 (IL-10) were determined in the culture supernatants from chondrocytes treated with different stimuli using commercial ELISA Kits (cat# KE1002, KE1003, Proteintech) following the manufacturer’s instructions. The absorbance at 450 nm was measured using a Multiskan Ascant (SPECTRAFluor Plus, Tecan).

### 2.9. Statistical analysis

The experiments were performed at least three times. The results are presented as the mean ± standard deviation. SPSS 13.0 software (SPSS Inc., Chicago, IL) was used to analyze the data and the results represent at least three independent experiments. Comparisons among multiple groups were performed using a one-way or two-way ANOVA followed by Tukey’s post hoc test and an unpaired Student’s *t* test between two groups. *P *< .05 was considered statistically significant.

## 3. Results

### 3.1. Curcumin alleviates inflammation in high glucose-induced PC12

**Figure 1.**
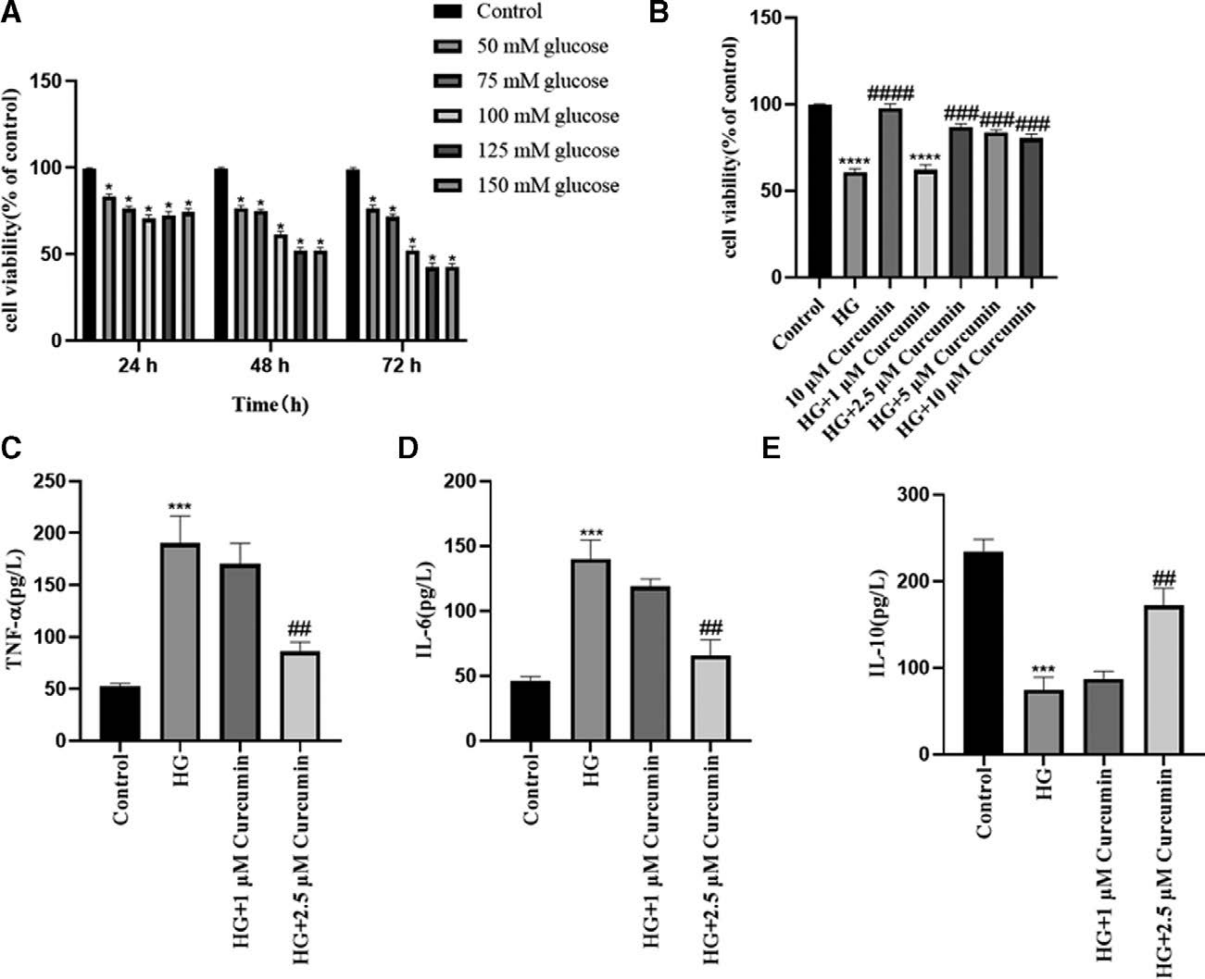
Effect of curcumin on HG-induced inflammation in neuronal PC12 cells. (A) Effect of HG administration for 24, 48, and 72 hours on neuronal PC12 cell viability. (B) Effect of curcumin on HG-induced cell toxicity in PC12 cells for 48 hours. Control; cells were incubated with normal glucose (5 mM); HG, cells were treated with HG (125 mM) medium; 10 μM curcumin, cells were treated with 10 μM curcumin in control medium; HG + curcumin, cells were pretreated with various concentrations of curcumin (1, 2.5, 5, and 10 μM) for 2 hours and then treated with HG (125 mM) medium. Cell viability was determined by the CCK8 assay. (C–E) PC12 cells were pretreated with 10 μM curcumin for 2 hours, followed by exposure to HG for 48 hours. The secretion of inflammatory cytokines, including tumor necrosis factor-α (TNF-α), interleukin-6 (IL-6), and interleukin-10 (IL-10) were detected by ELISA. The data are expressed as means ± SD; *P < .05, ****P < .0001 compared with Control and ^###^*P* < .001, ^####^*P *< .0001 compared with HG. HG = high glucose, PC12 cells = pheochromocytoma cells, SD = standard deviation, TNF-α = tumor necrosis factor-α.

The viability of PC12 cells was determined using the CCK8 assay to evaluate the effect of various HG concentrations. As shown in Figure 1A, PC12 cells were treated with normal glucose (5 mM) and high concentrations of glucose (50, 75, 100, 125, and 150 mM) for 24, 48, and 72 hours. The results indicated that 125 mM glucose decreased the viability of PC12 cells to 50% after 48 hours compared with the control (5 mM) cells. Toxicity was time-dependent and increasing the incubation time resulted in higher toxicity. Thus, 125 mM glucose for 48 hours was selected for subsequent experiments.

PC12 cells were pretreated with a corresponding concentration of curcumin (1–10 μM) for 2 hours, followed by exposure to 125 mM glucose (as high glucose) for 48 hours to establish the HG-induced cell model. The viability of PC12 cells was markedly decreased compared with the control group, and it was restored following treatment with 2.5 to 10 μM curcumin. Moreover, curcumin did not show any cytotoxicity toward PC12 cells (Fig. 1B). These results suggested that 2.5 μM curcumin protects PC12 cells from HG-induced cytotoxicity (Fig. 1B). Thus, 2.5 μM curcumin was selected for subsequent experiments.

To determine whether curcumin alleviates HG-induced cell injury, the levels of inflammatory factors were determined. As shown in Figure 1C–E, the ELISA results demonstrated that the levels of TNF-α and IL-6 were increased and those of IL-10 were decreased in the HG group compared with the control group, whereas treatment with curcumin resulted in the opposite effect (Fig. 1C–E). Overall, the aforementioned results suggest that curcumin attenuates inflammation in high glucose-treated PC12 cells.

### 3.2. miR-218-5p levels are increased and directly target TLR4 in high glucose-induced PC12 cells

**Figure 2.**
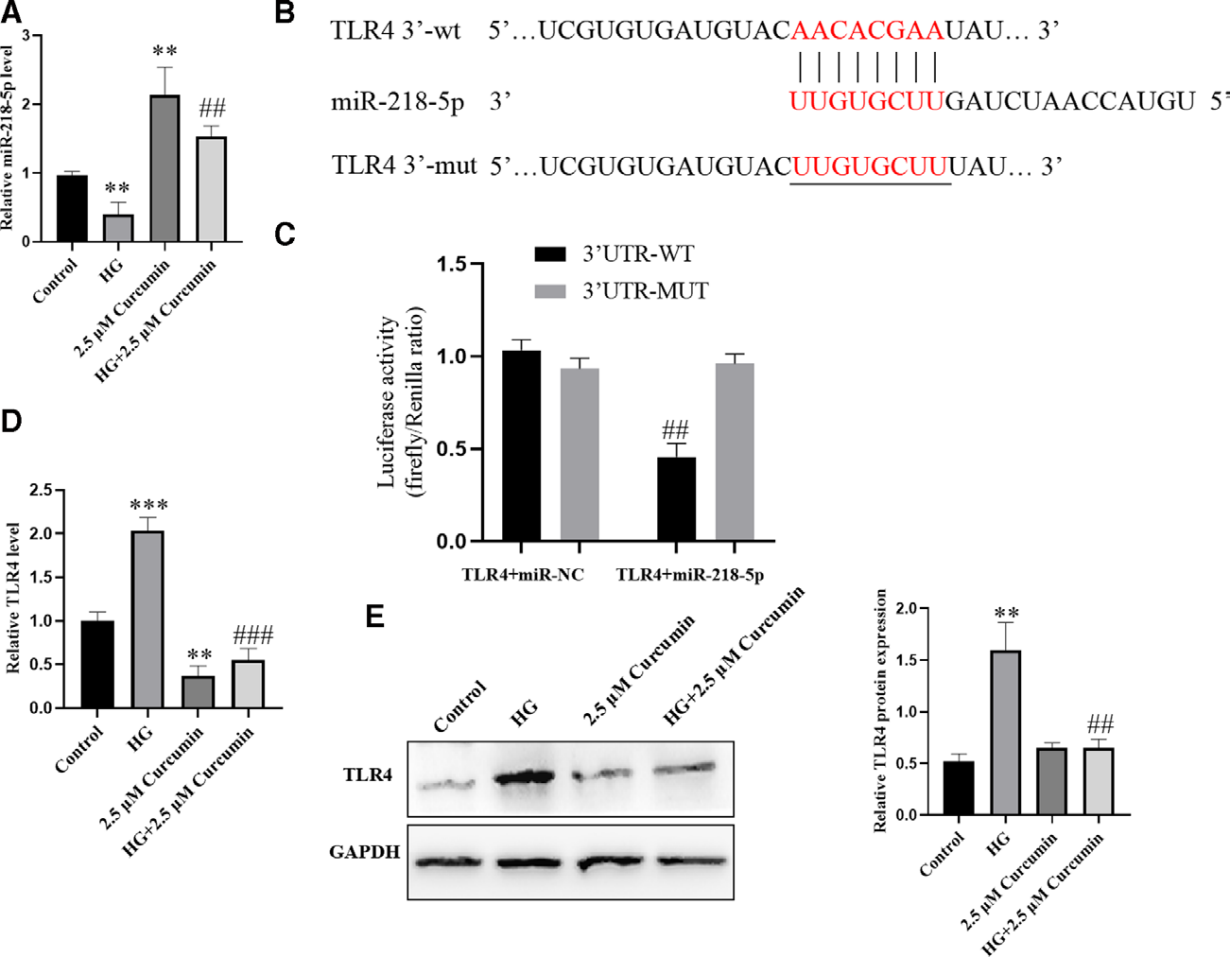
miR-218-5p levels are increased and directly target TLR4 in HG-induced PC12 cells. (A) miR-218-5p levels were measured by qRT-PCR. (B) The putative binding sites of miR-218-5p. (C) A luciferase reporter assay was used to investigate the interaction between miR-218-5p and TLR4. (D and E) The expression level of TLR4 was measured by qRT-PCR and Western blot analysis. The data are expressed as means ± SD; **P < .01, ***P < .001 versus Control. ^##^*P* < .01, ^###^*P* < .001 versus HG. HG = high glucose, PC12 cells = pheochromocytoma cells, qRT-PCR = quantitative real-time quantitative polymerase chain reaction, SD = standard deviation, TLR = toll-like receptors.

To assess whether the cytoprotective effect of curcumin against HG-induced toxicity was associated with miR-218-5p in PC12 cells, we evaluated the potential association between curcumin and miR-218-5p by qRT-PCR. We found that the levels of miR-218-5p were significantly decreased in the HG-treated group compared with the untreated group (Fig. 2A). In addition, treatment with curcumin enhanced the expression of miR-218-5p compared with the HG group. These results suggest that miR-218-5p may be activated by curcumin and involved in the neuroprotection of curcumin. Interestingly, the miRDB data and luciferase reporter assay results suggested that miR-218-5p directly targets the 3′-UTR of TLR4 to inhibit its expression (Fig. 2B and C). Subsequently, qRT-PCR and Western blot analysis were used to detect the expression of TLR4. In contrast, the expression of TLR4 was increased in HG-induced PC12 cells compared with controls and was decreased in the curcumin group compared with the HG group (Fig. 2D and E). Therefore, these results indicate miR-218-5p levels are increased and it directly targets TLR4 in HG-induced PC12 cells.

### 3.3. TLR4 overexpression blocks the suppressive effect of miR-218-5p downregulation on inflammation

**Figure 3.**
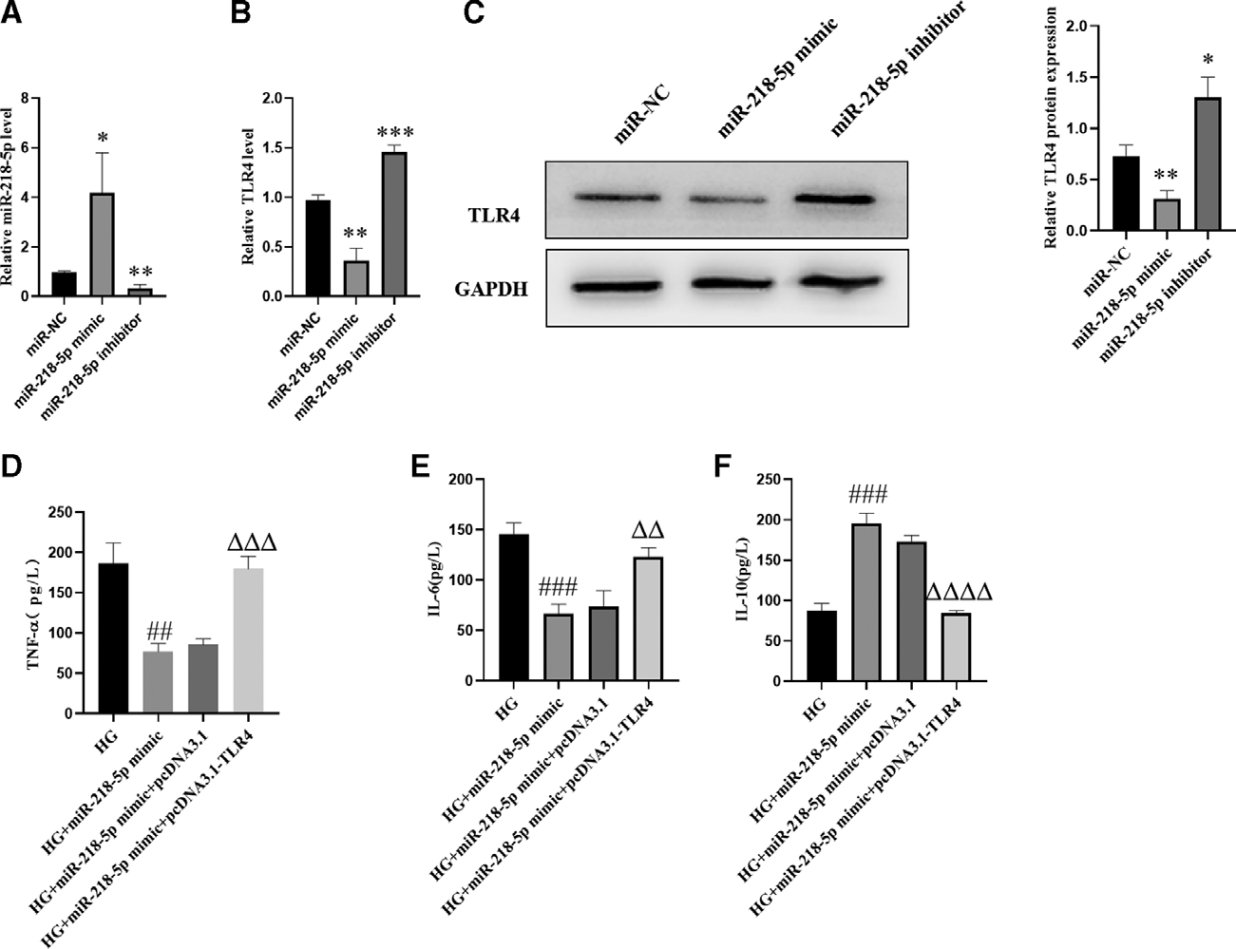
TLR4 overexpression blocks the suppressive effect of miR-218-5p downregulation on inflammation. (A) miR-218-5p levels were measured by qRT-PCR after transfection with miR-218-5p mimic and miR-218-5p inhibitor for 24 hours. (B and C) TLR4 expression was analyzed by qRT-PCR and Western blot analysis after transfection with miR-218-5p mimic and miR-218-5p inhibitor for 24 hours. (D–F) The secretion of inflammatory cytokines, including TNF-α, IL-6, and IL-10, were detected by ELISA. The data are expressed as means ± SD; *P < .05, **P < .01, ***P < .001, ****P < .0001 versus miR-NC. ^##^ *P* < .01, ^###^*P* < .001 versus HG. ^ΔΔ^*P* < 0.01, ^ΔΔΔ^*P* < 0.001, ^ΔΔΔΔ^*P* < 0.0001 versus HG + miR-218-5p mimic. ELISA = enzyme-linked immunosorbent assay, HG = high glucose, IL-6 = interleukin 6, IL-10 = interleukin-10, qRT-PCR = quantitative real-time quantitative polymerase chain reaction, SD = standard deviation, TLR4 = toll-like receptor 4, TNF-α = tumor necrosis factor-α.

To determine the underlying mechanism responsible for the cytoprotective effect of miR-218-5p in PC-2 cells, miR-218-5p mimic/inhibitor and TLR4 overexpression plasmids were generated to up- or down-regulate its expression respectively. The miR-218-5p mimic and inhibitor significantly increased and suppressed miR-218-5p expression levels, respectively (Fig. 3A). MiR-218-5p mimic significantly decreased TLR4 expression, whereas miR-218-5p inhibitor increased TLR4 expression at both the mRNA and protein level, respectively (Fig. 3B and C). ELISA assays were used to analyze the supernatants of HG-induced PC12 cells. The results indicated that the release of TNF-α and IL-6 were markedly increased, IL-10 were decreased in the HG group, the miR-218-5p mimic significantly inhibited the levels of TNF-α, IL-6, and increased the release of IL-10, which was reversed by TLR4 overexpression (Fig. 3D–F).

### 3.4. miR-218-5p silencing abrogates curcumin-ameliorated inflammation in high glucose-induced PC12 cells

**Figure 4.**
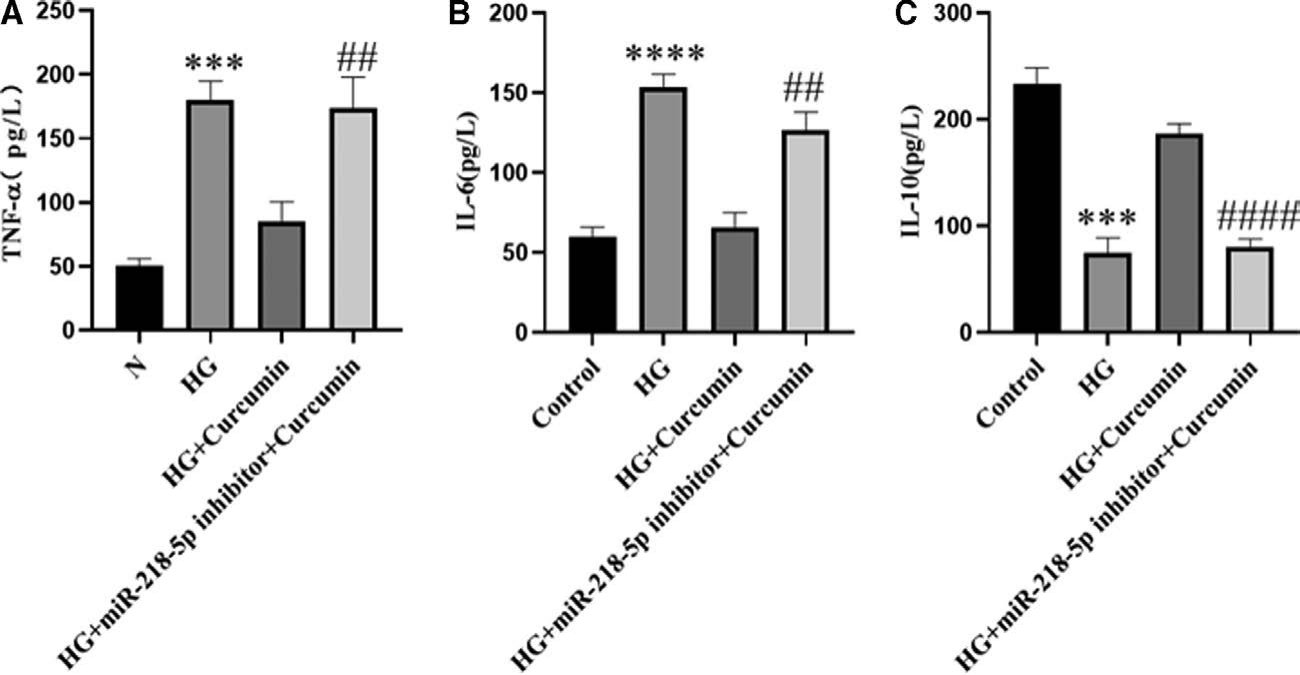
miR-218-5p silencing abrogates curcumin-ameliorated inflammation in HG-induced PC12 cells. (A–C) The secretion levels of inflammatory cytokines, including TNF-α, IL-6, and IL-10, were detected by ELISA. The data are expressed as means ± SD;***P < .001, ****P < .0001 versus Control. ^##^ *P* < .01, ^####^*P* < .0001 versus HG. ELISA-enzyme-linked immunosorbent assay, HG = high glucose, IL-6 = interleukin 6, IL-10 = interleukin-10, PC12 cells = pheochromocytoma cells, SD = standard deviation, TNF-α = tumor necrosis factor-α.

To further establish the role of miR-218-5p in the protective effects of curcumin, we pretreated cells with the miR-218-5p inhibitor prior to treatment with curcumin plus HG. The miR-218-5p inhibitor abolished the inhibition of TNF-α and IL-6 (Fig. 4A and B) and abolished the promotion of IL-10 in the presence of curcumin (Fig. 4C). These results indicate that the miR-218-5p was involved in the protective effects of curcumin.

## 4. Discussion

In the present study, we demonstrated the following^[1]^: treatment of PC2 cells with 125 mM HG for 48 hours resulted in viability inhibition and inflammation,^[2]^ HG-induced cell inflammation was decreased by pretreatment with curcumin,^[3]^ the anti-inflammatory effects of curcumin were mediated by the induction of miR-218-5p and a reduction of TLR4, and^[4]^ the miR-218-5p inhibitor abolished the curcumin-mediated downregulation of inflammatory factors.

Emerging evidence has indicated that inflammatory mechanisms play an important role in the pathogenesis and progression of DN, and may aggravate DN symptoms and increase the mortality of patients with DN.^[5,6]^ High glucose-induced neuronal inflammation is responsible for the development of diabetic neuropathy. PC12 cells, which are a mature cell line from a rat pheochromocytoma, are commonly used as a cell model for studying the mechanisms underlying glucose neurotoxicity,^[23]^ neuronal cell death, and neurotransmitter secretion.^[24]^ PC12 cells were also used to explore the protective effect of telmisartan on high glucose-induced neurotoxicity.^[25]^ However, the underlying mechanisms of how curcumin exerts its neuroprotective effect in high glucose-induced injuries remain unknown. Here we showed that curcumin suppresses PC2 cell inflammation. Curcumin is known for its anti-inflammatory and antioxidant activities. It suppresses IL-1β secretion and prevents inflammation through the inhibition of the NLRP3 inflammasome.^[26]^ More importantly, curcumin prevents brain damage and cognitive dysfunction during ischemic-reperfusion through the regulation of miR-7-5p.^[27]^ In isoflurane (ISO)-induced learning and memory dysfunction in Sprague-Dawley rats, curcumin exhibits a protective effect against ISO-induced cognitive dysfunction, which may be achieved by regulating the expression of miR-181a-5p.^[28]^ The related mechanistic studies show that curcumin reduces neuroinflammation and restores the impairment on learning and memory, which is consistent with our results.

It is well known that miRNAs are important molecular markers that may link obesity and neurodegeneration. Recent evidence has demonstrated the role of circulating levels of miRNAs in neurodegenerative disease.^[29–31]^ However, there have been few studies identifying the underlying mechanisms of miRNA transcriptional regulation in obesity, which causes cognitive decline and neurodegeneration. In the present study, we demonstrated a role for miR-218-5p and elucidated the underlying mechanisms that link HG with neurodegenerative disorders in the context of decreasing TLR4 expression. We found that when PC2 cells were treated with HG, the inflammatory response was markedly increased and miR-218-5p levels were decreased. Elevated miR-218-5p resulting from curcumin treatment may dramatically alleviate the levels of inflammatory factors induced by HG and play a protective role on cell inflammation. This protective effect may be achieved through miR-218-5p-targeted regulation of TLR4.

Toll-like receptors (TLRs) are an important class of transmembrane signaling molecules, which are known to activate the immune response during bacterial infection and cerebral injury.^[32]^ Of the TLRs, TLR4 plays an important role in the inflammatory response. Combined with its ligand, TLR4-induced nuclear factor kappa B (NF-κB) signaling pathway activation and inflammatory factor expression, such as cytokines, are upregulated to activate the immune response and inflammatory reaction.^[33]^ In the present study, there was statistical significance between the control group and the HG group, which proved that high glucose can upregulate TLR4 levels, which upregulates inflammatory factors and induces inflammation. However, miR-218-5p downregulated the expression of TLR4 and TLR4 overexpression blocked the suppressive effect of miR-218-5p downregulation on inflammation.

We found that miR-218-5p targeted TLR4, this causes the inhibition on inflammatory reaction induced by high glucose. If there are any miRNAs or target genes are involved in anti-inflammation effect is still unclear. So, the effect of other miRNAs in the anti-inflammation effect deserves further study. In addition to miRNAs, there are many peptidoglycans, proteins molecules, and other small peptides in curcumin. It is not clear whether there is a possible coactions relationship between the compounds in curcumin.

## 5. Conclusions

Above all, our study indicates that the induction of miR-218-5p represents a protective mechanism against inflammation during curcumin treatment in PC2 cells. This suggests that the anti-inflammatory effects of curcumin are mediated, at least in part, by regulating the miR-218-5p/TLR4 axis. This study provides a theoretical ground for the treatment of DN.

This work was supported by Scientific Research Foundation of Hebei Administration of Traditional Chinese Medicine (2021114).

## Author contributions

JXJ was responsible for the conception and design of the study. CY, SHT, WY, and ZP were involved in data acquisition. YX and WY was involved in the development of the study methodology, analysis and interpretation of the data. CY, SHT, ZP, WX, and JXJ were involved in the writing, reviewing and revision of the article, and analyzed the relevant literature. CY and JXJ confirmed the authenticity of the raw data. All authors have read and approved the final manuscript.

**Data curation:** Yuan Cui, Hong-Tao Song, Pei Zhang.

**Formal analysis:** Yuan Cui, Hong-Tao Song, Pei Zhang, Xiao Yin, Xuan Wei.

**Funding acquisition:** Yuan Cui, Xin-Ju Jia.

**Investigation:** Yuan Cui, Hong-Tao Song, Xiao Yin.

**Methodology:** Hong-Tao Song, Pei Zhang, Xiao Yin, Ying Wang.

**Project administration:** Xin-Ju Jia.

**Software:** Pei Zhang, Xiao Yin, Ying Wang, Xuan Wei.

**Supervision:** Xin-Ju Jia.

**Writing – original draft:** Yuan Cui, Xin-Ju Jia.

## Disclaimer

The authors declare that there is no conflict of interest regarding the publication of the paper.
